# Ibrutinib-Induced Skin Rash

**DOI:** 10.4274/tjh.galenos.2020.2020.0120

**Published:** 2021-02-25

**Authors:** Sudhir Kirar, Ajay Gogia, Ritu Gupta, Saumyaranjan Mallick

**Affiliations:** 1 New Delhi, India; 2 All India Institute of Medical Sciences, Medical Oncology, New Delhi, India; 3 Laboratory Oncology Unit, New Delhi, India; 4 All India Institute of Medical Sciences, Department of Pathology, New Delhi, India

**Keywords:** Ibrutinib, Skin rash, CLL

## To the Editor,

Ibrutinib is an oral irreversible inhibitor of Bruton’s tyrosine kinase (BTK), a B-cell receptor kinase that has been approved for use in chronic lymphocytic lymphoma (CLL), mantle cell lymphoma, and Waldenstrom macroglobulinemia. Cutaneous side effects of ibrutinib have been rarely reported and the most common presentation is skin rash [1]. We report an elderly patient with relapsed CLL who developed a severe skin rash within a week from the start of ibrutinib, which reappeared after the introduction of lower doses and required further discontinuation of drug.

**Figure 1:**
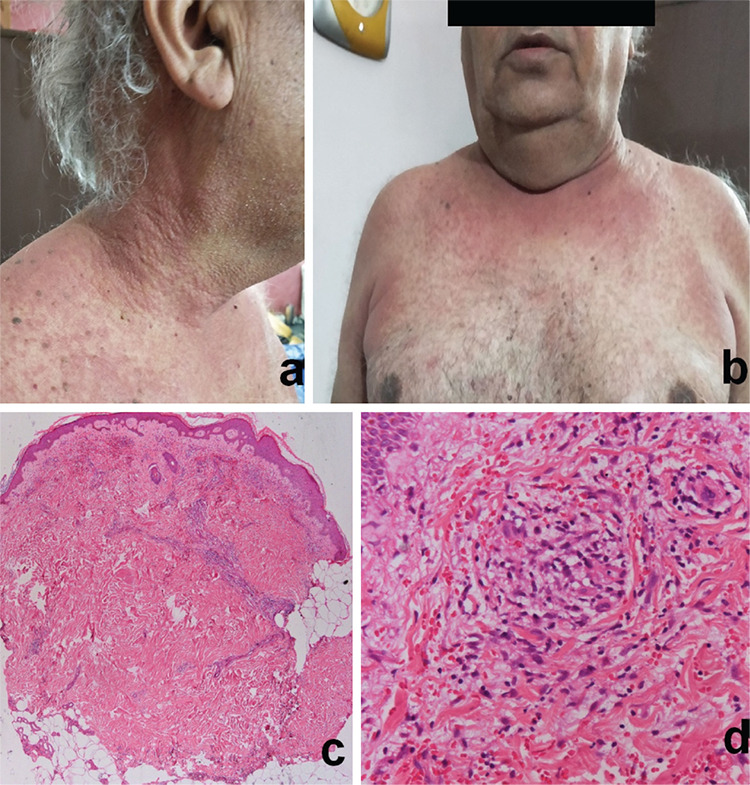
Severely itchy grade 3 maculopapular rash involving the nape of the neck, trunk, axilla, limbs, and groin area without any fever or symptoms of systemic allergy (a,b). Features suggestive of leukocytoclastic vasculitis (perivascular inflammatory exudates with extravasation of red blood cells) with elevated eosinophils consistent with drug eruption (c,d).

A 66-year-old obese male, previously diagnosed with CLL (RAI stage II), presented with rapid doubling of absolute lymphocyte count and fatigue after observation of 3 years. He had 13q deletion on fluorescent in situ hybridization. He also had comorbidities of hypothyroidism and idiopathic dilated cardiomyopathy with baseline left ventricular ejection fraction of 35%. He was started on ibrutinib at 420 mg once daily. He was concurrently receiving levothyroxine, aspirin, atorvastatin, and furosemide tablets for the last 10 years. On the fourth day after the start of ibrutinib, he developed severely itchy grade 3 maculopapular rash involving the nape of the neck, trunk, axilla, limbs, and groin area without any fever or symptoms of systemic allergy (Figures 1a and 1b). We attributed the rash as a side effect of ibrutinib because there were no confounding factors explaining the cutaneous findings. Ibrutinib was stopped and he was referred to a dermatologist. The skin rash responded to oral steroids and antihistamines with complete resolution by day 14. A punch biopsy specimen was taken and histopathological examination revealed features suggestive of leukocytoclastic vasculitis (perivascular inflammatory exudates with extravasation of red blood cells) with elevated eosinophils consistent with drug eruption (Figures 1c and 1d). Ibrutinib re-challenge was attempted with a dose of 140 mg once daily; however, he developed a similar grade 3 rash after 3 days. The rash disappeared 15 days after stopping ibrutinib and administration of systemic steroids necessitating permanent discontinuation of the culprit drug. At the last outpatient follow-up, the patient was doing well on infusion chemoimmunotherapy and the disease was in remission after 3 cycles.

Although ibrutinib is a highly selective BTK inhibitor, it exerts off-target effects on other kinases like epithelial growth factor receptor (EGFR) leading to inhibition of cell cycle progression and increased apoptosis. The inhibition of EGFR appears to be the most likely mechanism of ibrutinib-induced skin rash. Another proposed mechanism of ibrutinib-induced drug eruption is via inhibition of c-kit and platelet-derived growth factor receptor [2,3]. So far, few case reports, mostly in Western populations, have reported three types of ibrutinib-induced skin rash: a leukocytoclastic vasculitis-like pruritic violaceous palpable purpura, painless non-pruritic edematous papules with centripetal spread, and a third variety of asymptomatic non-palpable petechial rash [1,3]. Almost all of these were grade 1 or 2 in severity and were treated symptomatically with topical steroids and antihistamines without discontinuation of ibrutinib. To our knowledge, this is the first report from India where ibrutinib caused severe skin toxicity that required drug discontinuation and the mechanism is most probably due to hypersensitivity as there were abundant eosinophils in the histopathology specimen. With the availability of the generic form of ibrutinib in India and its increasing demand in cases of CLL, mantle cell lymphoma, and newer indications like chronic graft-versus-host disease, it is important to correctly identify and manage ibrutinib-induced skin toxicity.
